# Small Molecule Inhibitor of Type Three Secretion System Belonging to a Class 2,4-disubstituted-4H-[1,3,4]-thiadiazine-5-ones Improves Survival and Decreases Bacterial Loads in an Airway* Pseudomonas aeruginosa* Infection in Mice

**DOI:** 10.1155/2018/5810767

**Published:** 2018-09-10

**Authors:** Anna B. Sheremet, Naylia A. Zigangirova, Egor S. Zayakin, Sergei I. Luyksaar, Lydia N. Kapotina, Ludmila N. Nesterenko, Natalie V. Kobets, Alexander L. Gintsburg

**Affiliations:** Gamaleya Center of Epidemiology and Microbiology, Ministry of Health Russian Federation, 123098, Gamaleya Str. 18, Moscow, Russia

## Abstract

*Pseudomonas aeruginosa *is a cause of high mortality in burn, immunocompromised, and surgery patients. High incidence of antibiotic resistance in this pathogen makes the existent therapy inefficient. Type three secretion system (T3SS) is a leading virulence system of* P. aeruginosa *that actively suppresses host resistance and enhances the severity of infection. Innovative therapeutic strategies aiming at inhibition of type three secretion system of* P. aeruginosa *are highly attractive, as they may reduce the severity of clinical manifestations and improve antibacterial immune responses. They may also represent an attractive therapy for antibiotic-resistant bacteria. Recently our laboratory developed a new small molecule inhibitor belonging to a class 2,4-disubstituted-4H-[1,3, 4]-thiadiazine-5-ones, Fluorothiazinon (FT), that effectively suppressed T3SS in chlamydia and salmonella* in vitro *and* in vivo. *In this study, we evaluate the activity of FT towards antibiotic-resistant clinical isolates of* P. aeruginosa *expressing T3SS effectors ExoU and ExoS in an airway infection model. We found that FT reduced mortality and bacterial loads and decrease lung pathology and systemic inflammation. In addition, we show that FT inhibits the secretion of ExoT and ExoY, reduced bacteria cytotoxicity, and increased bacteria internalization* in vitro*. Overall, FT shows a strong potential as an antibacterial therapy of antibiotic-resistant* P. aeruginosa *infection.

## 1. Introduction 

*Pseudomonas aeruginosa *is an often cause of hospital pneumonia, urinary tract infections, primary bacteremia, and skin and soft tissue infections in burn, immunocompromised, and surgery patients [1–3]. It causes up to 34%-48% of all hospital infections with high mortality [4, 5]. Bacterial virulence factors affect host defenses and contribute to the immune misbalance favoring nonspecific inflammation and disturbing the initiation of protective responses towards pathogen [5, 6]. High incidence of multidrug-resistance in this pathogen adds to the severity of the situation.

Type three secretion system (T3SS) is a leading virulence system of pathogenic* Pseudomonas spp*. The proteins secreted by T3SS are toxins that induce cell apoptosis or necrosis, suppress the immune response, and inhibit macrophage and neutrophil recruitment and phagocytosis. In this context, innovative therapeutic strategies aiming at inhibition of T3SS activity are of particular interest, as they reduce the severity of clinical manifestations and improve antibacterial immune responses while preserving commensal flora [7]. These strategies also reduce the risk of selecting resistance, since they only disarm bacteria, allowing the host to employ immune mechanisms to fight the infection [8]. Different types of T3SS inhibitors are currently reported for Gram-negative bacteria such as therapeutic antibodies against* P. aeruginosa *PcrV protein [9], hybrid antibodies against PcrV and Psl [10], salicylidene acylhydrazides and hydroxyquinolines [11], and others [12, 13]. Recently our laboratory developed a new small molecule inhibitor designated as Fluorothiazinon (FT) that belongs to the class of 2,4-disubstituted-4H-[1,3, 4]-thiadiazine-5-ones. FT effectively suppressed T3SS in chlamydia and salmonella* in vitro *and* in vivo *[14–17]. FT significantly decreased mortality and bacteria loads in susceptible and resistant mice infected with* S. enterica serovar Typhimurium *[14]. FT inhibited the intracellular growth of different* Chlamydia *species in a dose-dependent manner and decreased the translocation of the type III secretion effector IncA [18]. FT possessed antibacterial activity* in vivo *and was able to control* C. trachomatis *serovar D vaginal shedding, ascending infection, and inflammation in the upper genital organs in DBA/2 mice [16]. Preclinical toxicological research confirmed its safety, lack of acute and chronic toxicity, mutagenicity, immunotoxicity, allergic potential, and lack of reproductive toxicity [14].

In this study, we evaluated the activity of FT towards antibiotic-resistant clinical isolates of* P. aeruginosa *expressing T3SS effectors ExoU and ExoS in an airway infection model. We found that FT reduced mortality and bacterial loads and decreased lung pathology and systemic inflammation. In addition, we showed that FT inhibited the secretion of ExoT and ExoY, reduced bacteria cytotoxicity, and increased bacteria internalization* in vitro*. Overall, FT shows a strong potential as an antibacterial therapy of antibiotic-resistant* P. aeruginosa *infection.

## 2. Material and Methods

### 2.1. Fluorothiazinon

Fluorothiazinon (FT) is N-(2,4-difluorophenyl)-4(3-ethoxy-4-hydroxybenzyl)-5-oxo-5,6-dihydro-4H-[1,3, 4]-thiadiazine-2 carboxamide previously reported as CL-55 and synthesized as described earlier [17]. For* in vitro *studies FT stock solution was prepared by dilution of 20 mg of FT, 44 mg of NaOH, and 77 mg of ammonium acetate in endotoxin-free deionized water, final pH – 7.0±0.2, to the final concentration of FT – 2 mg/ml.

### 2.2. Bacteria

*P. aeruginosa *clinical isolates used in this study were obtained from two Moscow hospitals and are listed in the Table S1 in Supplementary Materials.

### 2.3. Bacterial Culture

*P*.* aeruginosa *bacterial strains were streaked into LB broth from frozen stocks and grown overnight at 37°C. To assess FT antimicrobial activity* in vitro *night cultures were diluted in LB medium (1:100). FT was added to cultures to final concentrations of 5, 10, 20, and 40 *μ*g/ml. Gentamicin (Belmedpreparat, Minsk, Belarus) and Ciprofloxacin (CF) (Promed Exports, New Delhi, India) were added to final concentrations of 4 *μ*g/ml and 0.25 *μ*g/ml. Diluent without FT or antibiotics was used in the controls. Cultures were incubated overnight at 37°C with shaking. To assess the bacteria growth 10-fold serial dilutions of the cultures were seeded on Cetrimide agar (Cetrimide Agar Base w/o Glycerine, Himedia, Mumbai, India). Cultures were incubated at 37°С for 24 hours. The numbers of colonies were assessed as described elsewhere.

### 2.4. Mice and Ethics Statement

A/JsnYCit (A/Sn) mice were bred and maintained under conventional conditions at the Animal Facilities of the Gamaleya National Research Center for Epidemiology and Microbiology, Moscow, Russia, in accordance with National Guidelines. Male mice were used between 6 and 8 weeks of age. The Gamaleya National Research Center Animal Care Committee approved all experiments.

### 2.5. *In Vivo *Lung Infection Model

Two clinical isolates 1840 and KB6 with multiple antibiotic resistance were chosen for* in vivo *experiments (Supplementary Materials Table S1). Mice were infected intranasally with 40 *μ*l of* P. aeruginosa *culture in the doses indicated in the RESULTS section.

For* in vivo *experiments, FT was prepared by grinding of FT in 1% solution of starch. FT suspension was administered* per os *by gavage needle in a volume of 200 *μ*l. Mice received 50 mg/kg of FT twice a day for four days.

To assess the numbers of* P. aeruginosa *in lungs and spleens the specimens were homogenized in 1 ml of saline solution and centrifuged for 10 min at 800 rpm. 10-fold serial dilutions of organ homogenates were plated on Cetrimide agar and incubated for 24 hours at 37°С. Blood specimens were collected into tubes containing sodium heparin as an anticoagulant and 10-fold dilutions in saline were plated on Cetrimide agar.

### 2.6. Histochemistry

Lungs were sectioned and stained with hematoxylin and eosin as described before [16].

### 2.7. Cytokine Analysis

The concentrations of IL-6 and TNF-*α* in the blood and lung homogenates were determined using a commercial enzyme-linked immunosorbent assay kits (ELISA MAX Deluxe Set, Biolegend, San Diego, CA). Optical densities were measured using BioTek plate reader at the wavelength of 450 nm.

### 2.8. Immunoblot

Night cultures of* P. aeruginosa *were diluted 1:100 in a fresh LB medium with 5 mМ of EGTA and cultivated for 3 hours at 37°С. Bacteria were centrifuged and extracellular proteins were concentrated from supernatant by 10%-saturated trichloroacetic acid, washed with 100% acetone, resuspended in the sample buffer and subjected to a polyacrylamide gel electrophoresis as described previously [18]. After electrophoresis the proteins were transferred by a semidry blot from gel to nitrocellulose membranes using the TE70 PWR system (GE, Moscow, Russia) [19] Membranes were incubated with primary antibodies to ExoT and ExoY (in-house obtained mouse serum diluted 1:20000) overnight at 4°С. Blots were incubated with a secondary antibody linked to HRP (1:5000) for one hour at RT, and the signals were developed. The reaction was read with chemiluminometer (Vilber Lourmat, Eberhardzell Germany).

### 2.9. LDH Release Assay

Confluent CHO cells grown in RPMI-1640 medium supplemented with 10% fetal bovine serum (FBS) in 96-well plates were washed and covered with RPMI-1640 containing 1% FBS.* P. aeruginosa *grown overnight in LB medium was subcultured into fresh LB and grown to the mid-log phase. CHO cells were infected with the mid-log-phase* P. aeruginosa *at an initial multiplicity of infection (MOI) of 10. Plates were incubated for 3 hours in the presence of FT or diluent in the controls at the concentrations indicated in the RESULTS section. Plates were centrifuged at 1500 rpm for 10 min to sediment bacteria, and lactate dehydrogenase (LDH) release was measured in culture supernatants using CytoTox 96 nonradioactive cytotoxicity assay (Promega, Fitchburg, WI) in accordance with the manufacturer instructions. Percent of LDH release was calculated relative to the uninfected control, which was set as 0% of LDH release, and the cells lysed with Triton X-100, which was set as 100% of LDH release.

### 2.10. *Pseudomonas *Internalization Assay

*P. aeruginosa *was grown overnight in LB medium and further subcultured in fresh LB medium for 3 hours. After that, bacteria were washed and resuspended in DMEM with 1% of FBS. FT was added to* P. aeruginosa *at the concentrations indicated in the RESULTS section and incubated with shaking for 30 min. FT or diluent treated* P. aeruginosa *isolates were added to HeLa cells grown in 6-well plates at MOI of 10. After 2 hours of incubation extracellular bacteria were removed by washing with PBS, fresh DMEM medium containing 50 *μ*g/ml gentamicin was added, and cells were incubated for additional 2 h. After three washes with PBS, the cells were lysed in PBS containing 0.25% Triton X-100 and plated on the Cetrimide agar plates to count the number of bacteria internalized within HeLa cells.

### 2.11. Statistics

The results obtained from the mortality rates studies are represented as Kaplan-Meier survival curves, and the differences in survival were calculated by the log-rank test.

Significant differences of the other data were determined using the Mann–Whitney nonparametric two-tailed test using GraphPad Prism Version 6.

## 3. Results

### 3.1. Ft Promotes Survival of Animals in a Murine Model of* P. aeruginosa *Airway Infection Given Directly after the Onset of Infection

To investigate antibacterial effect of FT in the treatment of* P. aeruginosa *airway infection caused by antibiotic-resistant clinical isolates, we used* P. aeruginosa *clinical isolates of two different T3SS genotypes,* exoU*^+^ and* exoS*^+^ with multiple antibiotic resistance. A/Sn mice (n=10 per group) were infected intranasally with* exoU*^+^* P. aeruginosa *clinical isolate 1840 (6.5x10^6^ and 3.2x10^6^ CFU/animal) or* exoS*^+^* P. aeruginosa *clinical isolate KB6 (2.2x10^7^ and 1.1x10^7^ CFU/animal). Intranasal infection with* P. aeruginosa *clinical isolate 1840 in a dose of 6.5x10^6^ CFU/animal induced 80% of mortality (LD_80_) and 50% of mortality (LD_50_) in a dose of 3.2x10^6^ CFU/animal. Intranasal infection with* P. aeruginosa* clinical isolate KB6 in a dose of 2.2x10^7^ CFU/animal induced 90% of mortality (LD_90_) and 1.1x10^7^ CFU/animal induced 70% of mortality (LD_70_). The dose of FT and the regimen of treatment was evaluated in preliminary experiments (data is not shown). Infected animals were treated* per os *with 50 mg/kg of FT immediately after infection for 4 days twice a day, as this protocol was found the most effective (data is not shown).

**Figure 1:**
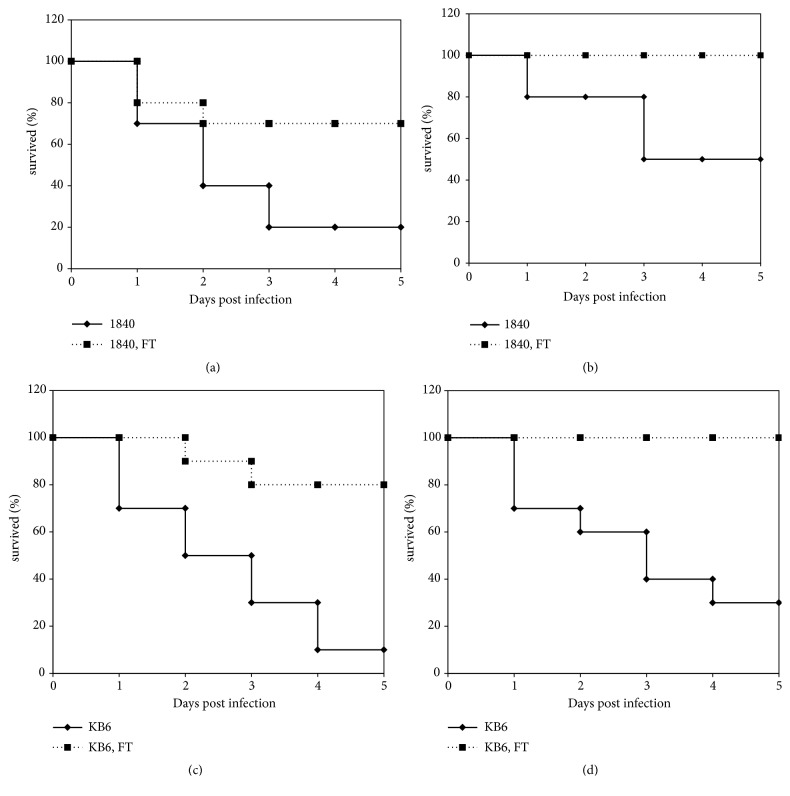
FT improves survival of mice infected with* P. aeruginosa *antibiotic-resistant clinical isolates of* exoU*^+^ (1840) and* exoS*^+^(KB6) genotypes. (a) mice infected with clinical isolate 1840, 6.45x10^6^ CFU/animal; (b) mice infected with clinical isolate 1840, 3.2x10^6^ CFU/animal; (c) mice infected with clinical isolate KB6, 2.2x 10^7^ CFU/animal; (d) mice infected with clinical isolate KB6, 1.1x10^7^ CFU/animal. Infected animals were treated with 50 mg/kg of FT* per os *immediately after infection for 4 days twice a day. Black line, control; dotted line, FT-treated mice.

As shown in Figure 1, FT provided survival of 70% of mice after infection with LD_80_ of clinical isolate 1840 (Figure 1(a)), and of 100% of mice after infection with LD_50_ (Figure 1(b)). FT protected 100% of animals after infection with LD_70_ of KB6 clinical isolate (Figure 1(d)) and 80% of animals after infection LD_90_ of the KB6 clinical isolate (Figure 1(c)). These results showed that FT administered* per os *reduced mortality of infected animals in the first 5 days postinfection.

### 3.2. FT Decreases Bacterial Loads in the Murine Model of* P. aeruginosa *Airway Infection Given Directly after the Onset of Infection

**Figure 2:**
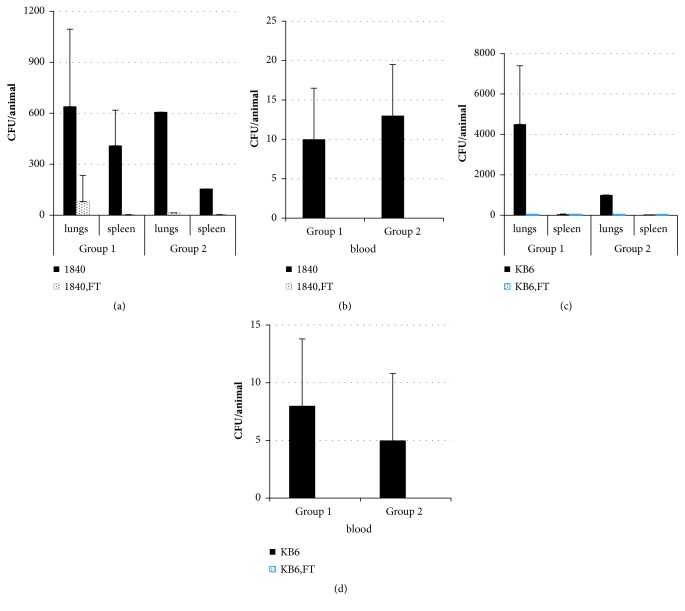
Treatment with FT reduces* P. aeruginosa *loads after infection with antibiotic-resistant* P. aeruginosa *clinical isolates of* exoU*^+^ (1840) and* exoS*^+^(KB6) genotypes. A/Sn mice were infected with 6.5х10^6^ CFU/mouse of* P. aeruginosa *clinical isolate 1840 ((a), group 1); with 3.25х10^6^ CFU/mouse of* P. aeruginosa *clinical isolate 1840 ((b), group 2); with 2.2x10^7^ CFU/mouse of* P. aeruginosa *clinical isolate KB6 ((c), group 1); with 1.1х10^7^ CFU/mouse of* P. aeruginosa*^+^ clinical isolate KB6 ((d), group 2). Infected animals were treated* per os *with 50 mg/kg of FT immediately after infection for 4 days twice a day. Bacterial loads in lungs, spleens, and blood of survived animals were analyzed at day 5 postinfection. Black bars, mice not treated with FT; dotted bars, mice treated with FT, P < 0.05.

Survived animals were sacrificed at day 5 postinfection. Average 6.4±4.5x10^2^ CFU/lung of clinical isolate 1840 was detected in the lungs of control animals infected with a dose of 6.5x10^6^ CFU/animal and average 6.08±7.6х 10^2^ CFU/lung was detected in lungs of animals infected with a dose of 3.25x10^6^ CFU/animal. Bacteria were found in spleens and blood of the control animals (Figure 2) that revealed systemic spread of infection. Administration of FT resulted in a decrease of bacterial burden by an order in lungs,and by two orders in the spleen compared to the controls (P≥ 0.05). Complete clearance of bacteria from the blood was observed in 40% of mice infected with 6.5x10^6^ CFU/animal and 100% of mice infected with 3.25x10^6^ CFU/animal of clinical isolate 1840.

For clinical isolate KB6 control mice infected with 2.2x10^7^ CFU/animal had 4.5±2.9 х 10^3^ CFU/lung and mice infected with 1.1x10^7^ CFU/animal had 1.4±1.08x10^3^ CFU/lung. Bacteria were also found in blood and spleens (Figure 2). Treatment with FT reduced bacterial loads in lungs. The numbers of bacteria decreased by two orders. 3 and 8 mice from high and low dose infection groups correspondingly were completely cleared from infection. All survived mice in the treated groups had no bacteria in the blood.

Thus, the results obtained in this study demonstrated the effectiveness of FT treatment in our airway infection model, induced with multiple antibiotic-resistant clinical isolates expressing various T3SS proteins. FT decreased mortality and bacterial loads in lungs and completely cleared infection from the blood.

### 3.3. FT Provided Survival of Animals in a Murine Model of* P. aeruginosa *Airway Infection Given as a Combined Prophylaxis-Treatment Regimen

*P. aeruginosa *clinical isolates 1840 and KB6 were used in this set of experiments. A/Sn mice (n=10 per group) were inoculated intranasally with* exoU*^+^* P. aeruginosa *cytotoxic clinical isolate 1840 with two doses: 7.0х10^6^ (LD_80_) and 3.5х10^6^ (LD_50_) CFU/mice; and* exoS*^+^* P. aeruginosa *clinical isolate KB6 in the doses of 1,75х10^7^ (LD_80_) and 8х10^6^ (LD_50_) CFU/animal.

**Figure 3:**
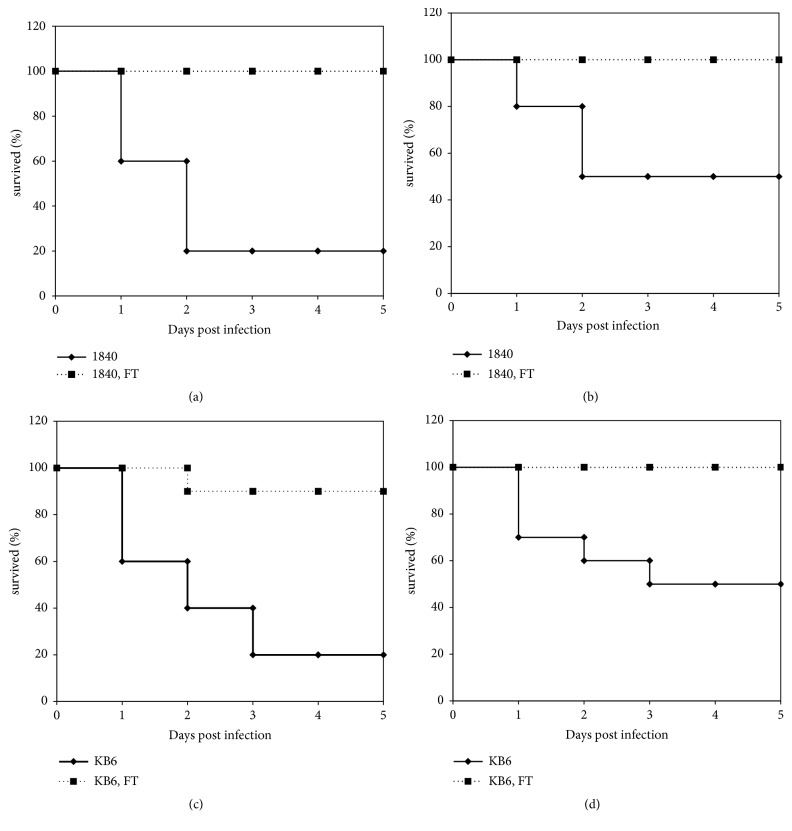
Combined prophylaxis-therapy treatment with FT improves survival of mice infected with* P. aeruginosa* antibiotic-resistant clinical isolates of* exoU*^+^ (1840) and* exoS*^+^(KB6) genotypes. (a) mice infected with clinical isolate 1840, 7.0х10^6^ CFU/animal; (b) mice infected with clinical isolate 1840, 3.5х10^6^ CFU /animal; (c) mice infected with clinical isolate KB6, 1.75х10^7^ CFU/animal; (d) mice infected with clinical isolate KB6, 8х10^6^ CFU/animal. Mice were treated with 100 mg/kg of FT* per os *once a day for 2 days before infection and with 50 mg/kg of FT twice a day for 4 days starting immediately after infection.

Mice were treated with 100 mg/kg of FT* per os *once a day for 2 days before infection and with 50 mg/kg of FT twice a day for 4 days starting immediately after infection. Survival rates and bacterial loads in lungs, spleens, and blood of survived animals were analyzed at day 5 postinfection. The results are presented in Figure 3. As shown in Figure 3, combined prophylaxis-therapy treatment with FT in mice infected with LD_80_ and LD_50_ of* P. aeruginosa exoU*^+^ clinical isolate 1840 led to 100% percent survival of animals. In the case of* P. aeruginosa exoS*^+^ KB6 infection (Figures 3(c) and 3(d)) the rate of survival was 90 and 100% for LD_80_ and LD_50_.

### 3.4. FT Decreased Bacterial Loads of Survivors in a Murine Model of* P. aeruginosa *Airway Infection Given as a Combined Prophylaxis-Treatment Regimen

**Figure 4:**
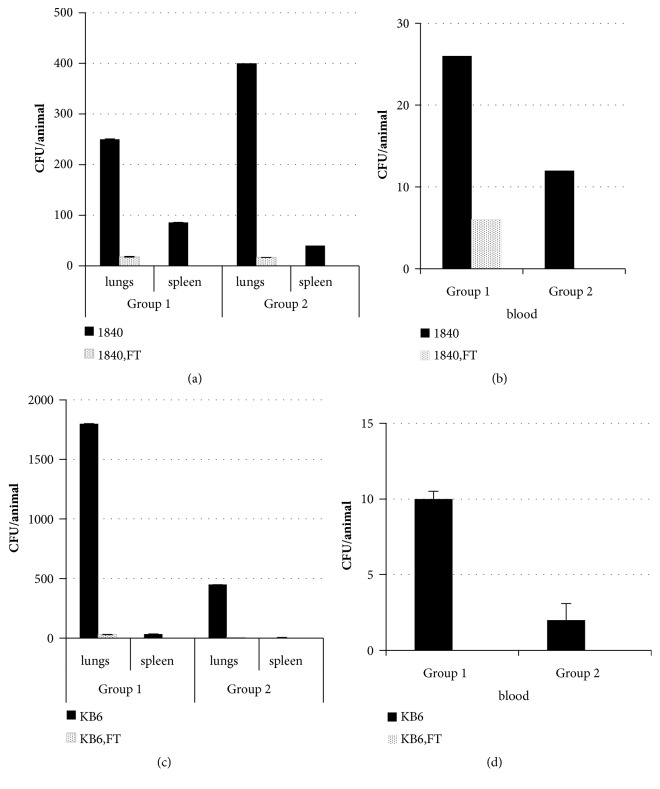
The prophylaxis-therapy regimen with FT reduces* P. aeruginosa *loads after infection with antibiotic-resistant* P. aeruginosa *clinical isolates of* exoU*^+^ (1840) and* exoS*^+^(KB6) genotypes. A/Sn mice were infected with 7x10^6^ CFU/mouse of* P. aeruginosa *clinical isolate 1840 ((a), group 1); with 3.5x10^6^ CFU/mouse of* P. aeruginosa *clinical isolate 1840 ((b), group 2); with 1.75x10^7^ CFU/mouse of* P. aeruginosa *clinical isolate KB6 ((c), group 1); with 8x10^6^ CFU/mouse of* P. aeruginosa *clinical isolate KB6 ((d), group 2). Mice were treated with 100 mg/kg of FT* per os *once a day for 2 days before infection and with 50 mg/kg of FT twice a day for 4 days starting immediately after infection. Bacterial loads in lungs, spleens, and blood of survived animals were analyzed at day 5 postinfection. Black bars, mice not treated with FT; dotted bars, mice treated with FT, P < 0.05.

To confirm eradication of bacteria, viable counts were performed on lung and spleen homogenates and blood from mice treated with FT. Survived animals were sacrificed at day 5 after the initiation of infection. The results are presented in Figure 4.

As shown in Figure 4, 2.5±0х10^2^ CFU/lung of clinical isolate 1840 was detected in lungs of control animals infected with a dose of 7x10^6^ CFU/animal and 3.4±4х10^2^ CFU/lung was detected in lungs of animals infected with a dose of 3.5x10^6^ CFU/animal. Infection was also found in spleens and blood (Figures 4(a) and 4(b)) that revealed the systemic spread of infection. Introduction of FT decreased bacterial burden in lungs, spleen, and blood compared to the controls (P≥ 0.05). Lungs of 70% of mice infected with 7x10^6^ CFU/animal of clinical isolate 1840 were cleared completely and all mice had no bacteria in spleen and blood. Statistically significant increase in the number of mice that cleared infection from lungs (8 compared to 1 in the control group) was found in the group of mice infected with 3.5x10^6^ CFU/animal of clinical isolate 1840. For the clinical isolate KB6 mice infected with 1.75x10^7^ CFU/animal had 1.8±0х10^3^ CFU/lung of bacteria and mice infected with 8x10^6^ CFU/animal had 4,7±3,6х10^2^ CFU/lung of bacteria. Infection was also found in blood and spleen (Figures 4(c) and 4(d)). Treatment with FT reduced bacterial loads in lungs (2.1±3.4х10^1^ and 0.6±1.0х10^1^ correspondingly). 7 and 9 mice from high and low dose infection groups correspondingly were completely cleared from infection. Survived mice had no bacteria in the blood.

Therefore, the results obtained give the evidence on the effectiveness of FT in the combined prophylaxis-treatment regimen of airway infection, induced with multiple antibiotic-resistant clinical isolates expressing various T3SS proteins. FT decreased mortality and bacterial loads in lungs and completely cleared infection from the blood.

### 3.5. Reduced Lung Damage after Treatment with FT

**Figure 5:**
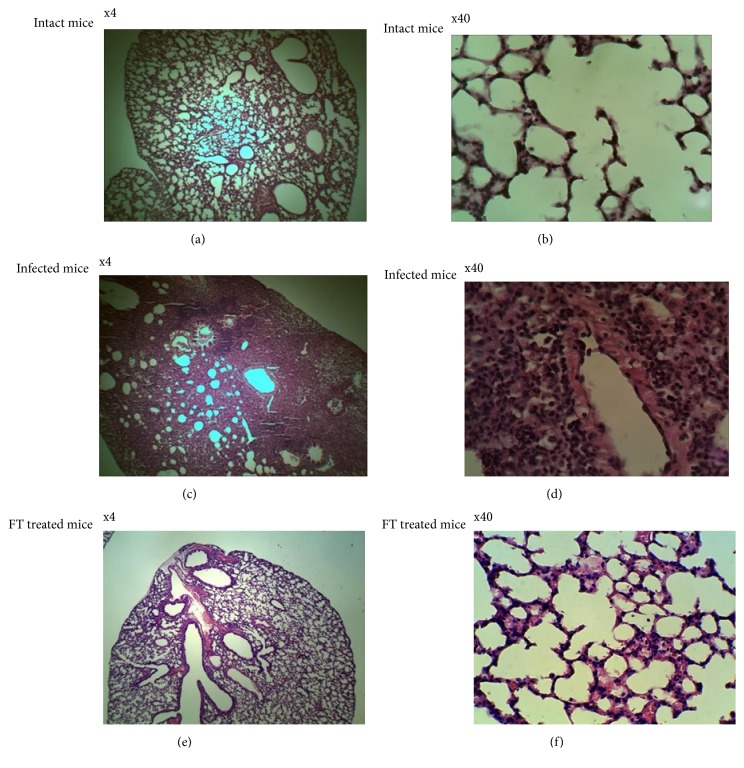
FT reduces lung pathology in mice intranasally infected with* P. aeruginosa *clinical isolate* exoU*^+^ 1840 in a dose of 3.25х10^6^ CFU/mouse. Mice were treated* per os *with 50 mg/kg of FT twice a day for 3 days starting immediately after infection. Lungs of survived animals were sectioned, stained with H&E and analyzed at day 5 postinfection. Photographs were taken at multiplication x4 (a, c, e) and x40 (b, d, f) for intact (a, b); infected (c, d) and FT-treated mice (e, f).

Lung morphology was studied in mice infected with 3.25x10^6^ CFU/animal of* exoU*^+^ clinical isolate 1840 and treated with FT. To this end, mice were treated* per os *with 50 mg/kg of FT twice a day for 3 days. The results are presented in Figure 5. Infection resulted in a pronounced damage to lungs as deduced from H&E staining. Damaged alveoli structure, peribronchial leukocyte infiltration, and dense parenchyma indicated severe lung inflammation (Figures 5(c) and 5(d)). Treatment with FT resulted in decreased cellularity in alveoli and in interstitial spaces and alveolar septal thickening. However, separate spots of infiltration were still observed in the treated groups (Figures 5(e) and 5(f)). Overall, these results suggest that FT effectively prevents lung damage in mice infected with multiple antibiotic-resistant clinical isolates of* P. aeruginosa*.

### 3.6. FT Modulates Proinflammatory Cytokines in the Course of* P. aeruginosa *Airway Infection

**Figure 6:**
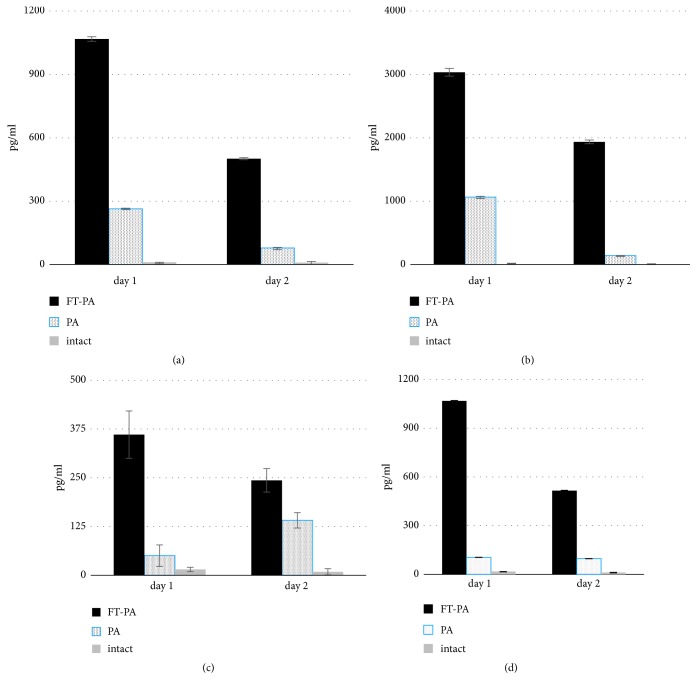
FT increases the production of inflammatory cytokines in lungs but decreases IL-6 production in blood. A/Sn mice were infected intranasally with 10^7^ CFU/animal of* P. aeruginosa exoS*^+^ clinical isolate KB6. Mice were treated with 50 mg/kg of FT* per os *twice daily before infection and once postinfection. IL-6 (a), TNF-alpha (b), and IFN-gamma (c) were tested in lung homogenates at day 1 and 2 PI. IL-6 (d) in blood was tested at day 2 PI. Black bar, treatment with FT; dotted bar, untreated infected mice; grey bar, intact controls, P < 0.05.

In this study, we have analyzed the effect of FT treatment on cytokine production in lungs and blood during* P. aeruginosa *airway infection induced by 10^7^ CFU/animal of* exoS*^+^ clinical isolate KB6. Mice were treated* per os *with 50 mg/kg of FT twice daily before infection and once postinfection. Mice were sacrificed 24 and 48 hours after bacterial infection (n=10 in each group, two separate experiments). The levels of key cytokines involved in the regulation of inflammation were determined in lung tissue homogenates and blood serum using ELISA (Figure 6).

Treatment with FT significantly increased the levels of the proinflammatory cytokines IL-6 and TNF-alpha and IFN-gamma in lung homogenates at day 1 postinfection (Figures 6(a), 6(b), and 6(c)). The levels of these cytokines were also higher in FT-treated group compared to nontreated mice at day 2 postinfection, however, tended to decrease compared to the levels at day 1.

In contrast, in blood, we observed a significant decrease of IL-6 at day 2 postinfection in FT- treated mice infected with KB6 compared to nontreated animals (Figure 6(d)). No alterations of TNF-alpha or IFN-gamma were observed in the blood of all experimental groups compared to controls (data is not shown).

Generally, the effects from FT were seen in all compartments investigated, suggesting both systemic effects and effects within the lung. FT increased the levels of proinflammatory cytokines in lungs at early stages of infection that probably reflects its ability to confront virulence mediated downregulation of host defenses; however, it significantly decreased the level of systemic production of IL-6 in blood that in line with a decrease in systemic bacterial loads manifests its potential to control systemic infection.

### 3.7. FT Inhibits Secretion of* P. aeruginosa *T3SS Effectors and Bacteria Cytotoxicity and Restores Bacterial Internalization

**Figure 7:**
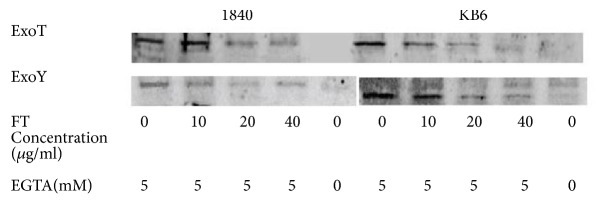
FT inhibits the secretion of* P. aeruginosa *T3SS effectors in the clinical isolates under the study. Expression of T3SS was induced by adding 5 mM of EGTA to culture media. FT was added in concentrations from 10 to 40 *μ*g/ml. ExoT and ExoY were evaluated by immunoblot with polyclonal antibodies against ExoТ and ExoY.

Next, we have assessed the effects of FT on the secretion of T3SS effectors. To this end, we evaluated* in vitro *secretion of ExoT and ExoY proteins in* P. aeruginosa *clinical isolates by immunoblot. Clinical isolates 1840 and KB6 were incubated* in vitro *with different concentrations of FT for 4 hours. Expression of T3SS was induced by decreasing Са^+2^ concentration. We have found that FT inhibits secretion of ExoT and ExoY in a dose-dependent manner as shown in Figure 7. Inhibition of T3SS in clinical isolates 1840 and KB6 was observed starting with the concentration of 10 *μ*g/ml. No difference in bacterial growth in the presence or absence of FT for* P. aeruginosa *reference strains as well as for clinical isolates cultured for 24 hours was observed in these experiments (Supplementary Materials, Table S2). Thus, we confirmed that FT downregulates the secretion of* P. aeruginosa *T3SS effector proteins.

**Figure 8:**
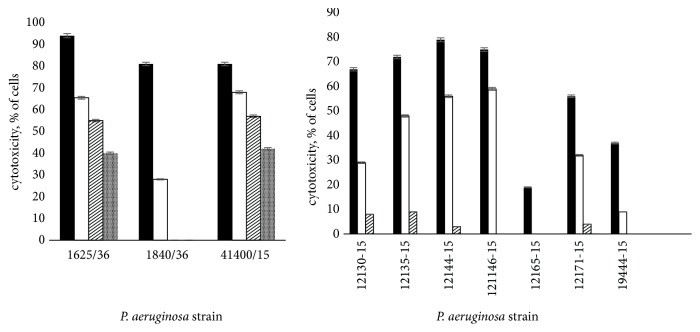
FT reduced* P. aeruginosa *cytotoxicity towards CHO cells. (a) ExoU expressing* P. aeruginosa *clinical isolates; (b) ExoS expressing* P. aeruginosa *clinical isolates. Dark bars, controls; white bars, 10 *μ*g/ml of FT; crossed bars, 20 *μ*g/ml; checkered bars, 40 *μ*g/ml of FT, P < 0.05.

**Figure 9:**
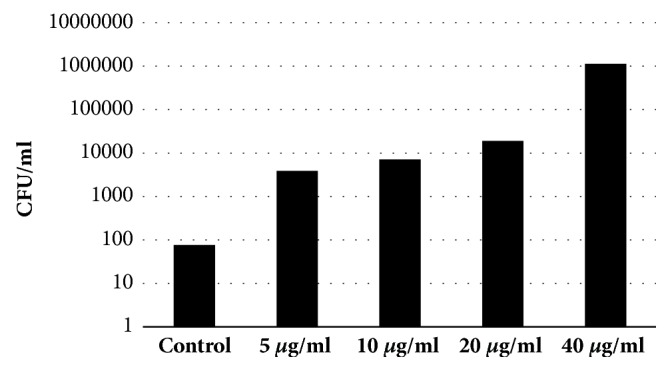
FT increased bacteria internalization in a dose-dependent manner. To assess the capability of FT to affect bacteria internalization,* P. aeruginosa exoS*^+^ clinical isolate 1653, sensitive to gentamicin, was preincubated with FT for 30 min and was added to HeLa cells at MOI of 10. After incubation for 2 hours extracellular bacteria were eliminated by gentamicin. The numbers of intracellular bacteria were determined 2 hours later, P < 0.05.

Next, we assessed the effects of FT on* P. aeruginosa *induced cytotoxicity. CHO cells were infected with* P. aeruginosa *clinical isolates preliminary incubated for 30 minutes with different concentrations of FT. We found that* P. aeruginosa *clinical isolates induced profound cell cytotoxicity given in a dose of 10 MOI. Addition of FT in the doses of 10, 20, and 40 *μ*g/ml significantly reduced cell cytotoxicity (P≤ 0.05). The results are presented in Figure 8. We found that FT inhibited cytotoxicity of* P. aeruginosa *clinical isolates in a dose-dependent manner. We found that FT completely inhibited cytotoxicity of ExoU expressing clinical isolate 1840 at the concentration of 20 *μ*g/ml. The cytotoxicity of two other* exoU*^+^ isolates was inhibited up to 50%. FT inhibition of ExoS expressing* P. aeruginosa *clinical isolates cytotoxicity was more pronounced compared to ExoU expressing strains.* P. aeruginosa *ExoS and ExoT were shown to prevent bacteria internalization by epithelial and phagocytic cells that in turn reduces bacteria elimination by phagocytes and facilitates the spread of infection. To assess the capability of FT to affect bacteria internalization,* P. aeruginosa *clinical isolates were preincubated with FT for 30 min and were added to HeLa cells at MOI of 10. After incubation for 2 hours extracellular bacteria were eliminated by gentamicin. The numbers of intracellular bacteria were determined 2 hours later.* P. aeruginosa exoS*^+^ clinical isolate 1653, sensitive to gentamicin, was used in these experiments. We have found that FT increased bacteria internalization in a dose-dependent manner (Figure 9). Even 5 *μ*g/ml gave a 50-fold increase in the quantity of internalized bacteria, while 40 *μ*g/ml of FT gave 10^4^ increase in bacteria internalization.

## 4. Discussion

This study suggests that a small molecule compound, designated as Fluorothiazinon (FT), given as a combined prophylaxis-therapy treatment or as a therapy started after the onset of infection may improve the outcome in severe antibiotic-resistant* P. aeruginosa *airway infection.

Therapeutic agents that target virulence determinants of pathogenic bacteria have become an increasingly promising alternative to antibiotics [20, 21]. T3SS proteins are attractive targets for “anti-virulence” compounds because they are often essential to the virulence of widely distributed Gram-negative bacterial pathogens of plants, animals, and humans. Targeting only virulence and lacking unwanted side effects such as evolvement of antibiotic-resistant variants makes this therapeutic strategy highly promising. Recently, whole-cell-based high-throughput screens performed to identify T3SS inhibitors gave several classes of small molecule compounds. Salicylidene acylhydrazides, salicylanilides, sulfonylaminobenzanilides, benzimidazoles, thiazolidinone, and some natural products were shown to be effective against a number of pathogenic bacteria that utilize T3SS, including* Yersinia, Chlamydia, Salmonella*, enteropathogenic* Escherichia coli, Shigella*, and* Pseudomonas* [11, 22–25].

A novel compound with a predicted T3SS inhibitory activity named FT, N-(2,4-difluorophenyl)- 4-(3-ethoxy-4-hydroxybenzyl)-5-oxo-5,6-dihydro-4H-[1,3, 4]-thiadiazine2-carboxamide was previously characterized by low toxicity, high levels of solubility, stability, and specific efficiency towards* C. trachomatis *and* Salmonella in vitro *and* in vivo *[14, 16]. Besides, FT was shown to decrease the translocation of the type III secretion effector IncA of* C. trachomatis *[17].

In this study, we show that FT given as a combined prophylaxis-therapy treatment regimen or as a therapy started after the onset of infection significantly reduced mortality of mice infected with antibiotic-resistant* P. aeruginosa *clinical isolates. Besides, FT treatment significantly reduced bacterial loads in lungs and blood of experimental animals. The potential of FT to control generalized pseudomonas infection induced by antibiotic-resistant clinical isolates can be of great importance for improved clinical outcomes.

Lung infection with* P. aeruginosa *antibiotic-resistant clinical isolates of two different T3SS genotypes in our model was associated with severe lung inflammation reflected as damaged alveoli structure, peribronchial leukocyte infiltration, and parenchyma thickening. This represents an important feature of* Pseudomonas *lung pathogenesis and in line with clinical data. FT treatment resulted in the rehabilitation of alveoli structure and lesser interstitial cellularity (Figure 5); however, residual leukocyte infiltration of lungs was still observed in FT-treated groups.

Therefore improved mortality rates and decreased bacterial loads were associated with a decrease of lung pathology as analyzed at day 5 postinfection. That might be due to the lesser numbers of bacteria in lungs as shown in other studies [26]. Furthermore, diminished virulence of bacteria due to downregulation of exotoxins (Figure 7) can also contribute to the decrease in lung pathology [27].

The dysregulated host responses to bacterial toxins are of critical importance during severe infections [28]. T3SS was shown to interfere with the protective host responses. Thus,* P. aeruginosa *T3SS effector protein ExoU can inhibit activation of the NLRC4 inflammasome and caspase-1 and, as a result, downregulates rapid neutrophil recruitment and rapid infection clearance [29]. ExoS was shown to prevent neutrophil recruitment and efficient clearance of bacteria [30]. As neutrophil accumulation in lungs is under the control of tumor necrosis factor-alpha (TNF-alpha) [31] in the present study we evaluated its production in lungs of mice infected with antibiotic-resistant clinical isolates 1840 and KB6. IL-6 and IFN-gamma were also evaluated in this study. Treatment with FT significantly increased the levels of IL-6, IFN-gamma, and TNF-alpha in lungs at day 1 postinfection compared to nontreated animals. The quantity of IL-6, IFN-gamma, and TNF-alpha in FT-treated group decreased at 48 hours postinfection but still remained higher than in infected group not treated with FT. IL-6 in lungs of pseudomonas-infected animals was recently shown to contribute to the local protection against some of* P. aeruginosa *toxins [32]. At the same time, it was found to be harmful in generalized infection and sepsis [33]. In our study in contrast to elevated levels of IL-6 levels in lungs, blood IL-6 levels were decreased in FT-treated group compared to nontreated infected mice. These data suggest FT potential to control generalization of inflammatory processes along with generalized infection. This is also in line with the general concept of the roles of T3SS in the dissemination of infection [34]. Our results on reducing of lung pathology in FT-treated mice suggest that FT decreases the severity of infection induced by antibiotic-resistant* P. aeruginosa *resistant clinical isolates. In this study, we also assessed FT effects on* P. aeruginosa *T3SS toxins as it was originally described as inhibitors of T3SS in* Salmonella *and* Chlamydia *spp. [14–17]. We found that FT reduces the total amount of ExoT and ExoY toxins detected by Western blot in FT-treated cultures of* P. aeruginosa *clinical isolates 1840 and KB6 (Figure 7). Besides, FT increased bacteria internalization in HeLa cells (Figure 9) and reduced cytotoxicity of various* P. aeruginosa *clinical isolates towards CHO cells (Figure 8). Our results on FT activity on the secretion of T3SS effectors and their functions are in line with previously reported data for different T3SS inhibitors. Thus, the inhibition of the T3SS-mediated secretion and translocation of ExoS or ExoT by mutation was shown to increase internalization of bacteria (6, 15, 18, 50). Overall, the results obtained in this study suggest that FT is a promising novel T3SS inhibitor of pulmonary antibiotic-resistant* P. aeruginosa *infection.

## 5. Conclusions

In conclusion, in this study we found that Fluorothiazinon successfully reduced mortality and bacterial loads and decreased lung pathology and systemic inflammation in a mouse bronchopulmonary model. It inhibited the secretion of T3SS effectors ExoT and ExoY, reduced bacteria cytotoxicity, and increased bacteria internalization* in vitro*. Overall, FT shows a strong potential as an antibacterial therapy of antibiotic-resistant* P. aeruginosa *infection.

## Data Availability

For more data from the article, please send a request to the mail snejpice@gmail.com, Anna Sheremet.

## Supplementary Materials

Supplementary 1 Table S1.* P. aeruginosa* clinical isolates.

Supplementary 2 Table S2. Impact of FT on* P. aeruginosa* clinical isolates growth.
