# Supplementary material for: Small Molecule Inhibitor of Type Three Secretion System Belonging to a Class 2,4-disubstituted-4H-[1,3,4]-thiadiazine-5-ones Improves Survival and Decreases Bacterial Loads in an Airway Pseudomonas aeruginosa Infection in Mice
Source: Biomed Res Int. 2018 Sep 10;2018:5810767. doi: 10.1155/2018/5810767 (PMC6151375; doi:10.1155/2018/5810767)
Supplement: Supplementary 1 — Table S1. P. aeruginosa clinical isolates. [file 5810767.f1.docx]

| **Table S1. *P. aeruginosa* clinical isolates.** | | | | | | | | | |
| --- | --- | --- | --- | --- | --- | --- | --- | --- | --- |
|  | Clinical isolate and its origin | *exoT* | | | *exoY* | *exoU* | | *exoS** | |
| 1. | **PAO103** | + | | | + | + | | - | |
| Resistant to: | Ticarcillin, meropenem |  | | |  |  | |  | |
| Susceptible to: | Carbenicillinum, piperacillin/tazobactam, gentamicin, aztreonam, meropenem, imipenem, levofloxacin, ceftazidime, cefipim, tobramycin | | | | | | | | |
| 2. | **PAO1** | | + | + | | | - | | + |
| Resistant to: | n/a | | | | | | | | |
| Susceptible to: | Piperacillin, aztreonam, ceftazidime, meropenem, gentamicin, tobramycin, amikacin, colistin, co-trimoxazole | | | | | | | | |
| 3. | **КБ6/6/2014,** bronchoalveolar lavage | + | | | + | - | | + | |
| Resistant to: | n/a | | | | | | | | |
| Susceptible to: | Aztreonam, colistin, piperacillin-tazobactam | | | | | | | | |
| 4. | **1840/36/2015,** burn wound. | + | | | + | + | | - | |
| Resistant to: | Amikacin, gentamicin, imipenem/cilastatin, levofloxacin, meropenem, piperacillin, a  piperacillin/tazobactam, tobramycin, cefazolin, cefipim, cefoperazone/sulbactam, cefoxytin, ceftazidime, ceftriaxone, ciprofloxacin. | | | | | | | | |
| Susceptible to: | colistin | | | | | | | | |
| 5. | **41400/15/2015**, wound, osteomyelitis | + | | | + | + | | - | |
| Resistant to: | Aztreonam, amikacin, ampicillin, ampicillin/sulbactam, gentamicin, co-trimoxazole (biseptol), meropenem, netilmicin, piperacillin/tazobactam, tigecycline, cephalexin,  cefipim, cefotaxime, ceftazidime, ciprofloxacin. | | | | | | | | |
| Susceptible to: | colistin | | | | | | | | |
| 6. | **12130m/15/2015**,  sputum cystic fibrosis | + | | | + | - | | + | |
| Resistant to: | Ceftriaxone | | | | | | | | |
| Susceptible to: | Amikacin, gentamicin, imipenem/cilastatin, levofloxacin, meropenem, polymyxin B,  tobramycin, cefipim, cefoperazone, ceftazidime, ciprofloxacin | | | | | | | | |
| 7. | **19444/15/2015,** bronchoalveolar  lavage, intense therapy. | + | | | + | - | | + | |
| Resistant to: | Aztreonam, amikacin, Amoxicillin/clavulanic acid, ampicillin, gentamicin, imipenem/cilastatin, co-trimoxazole (biseptol), levofloxacin, meropenem, piperacillin, piperacillin/tazobactam, tigecycline, tobramycin, cefipim, cefotaxime, ceftazidime,  ceftriaxone, cefuroxime, ciprofloxacin | | | | | | | | |
| Susceptible to: | Colistin | | | | | | | | |
| 8. | **12135/15/2015**, sputum, cystic  fibrosis. | + | | | + | - | | + | |
| Resistant to: | Ceftriaxone | | | | | | | | |
| Susceptible to: | Amikacin, gentamicin, imipenem/cilastatin, levofloxacin, meropenem, polymyxin B,  tobramycin, cefipim, ceftazidime, cefoperazone, ciprofloxacin | | | | | | | | |
| 9. | **12144/15/2015**, sputum, cystic  fibrosis. | + | | | + | - | | + | |
| Resistant to: | Amikacin, gentamicin | | | | | | | | |
| Susceptible to: | Imipenem/cilastatin, levofloxacin, meropenem, polymyxin B, tobramycin, cefipim,  cefoperazone, ciprofloxacin, ceftazidime | | | | | | | | |
| 10. | **12146/15/2015**, sputum, cystic  fibrosis. | + | | | + | - | | + | |
| Resistant to: | Amikacin, gentamicin | | | | | | | | |
| Susceptible to: | Piperacillin, tazobactam, polymyxin В, polymyxin Е (colistin), cefipim, ceftazidime,  ciprofloxacin | | | | | | | | |
| 11. | **12165/15/2015**, sputum, cystic  fibrosis. | + | | | + | - | | + | |
| Resistant to: | Ceftriaxone | | | | | | | | |
| Susceptible to: | Amikacin, gentamicin, imipenem/cilastatin, meropenem, polymyxin В, polymyxin Е  (colistin), tazobactam, tobramycin, cefipim, cefoperazone, ceftazidime | | | | | | | | |
| 12. | **12171/15/2015**, sputum, cystic  fibrosis. | + | | | + | - | | + | |
| Resistant to: | Meropenem, ceftriaxone, cefoperazone | | | | | | | | |
| Susceptible to: | Amikacin, gentamicin, imipenem/cilastatin, polymyxin В, polymyxin Е (colistin),  tobramycin, cefipim, ceftazidime, ciprofloxacin. | | | | | | | | |
